# Higher albumin levels on admission predict better prognosis in patients with confirmed COVID-19

**DOI:** 10.1371/journal.pone.0248358

**Published:** 2021-03-16

**Authors:** Matthew Kheir, Farah Saleem, Christy Wang, Amardeep Mann, Jimmy Chua

**Affiliations:** 1 Department of Medicine, Trios Health- A University of Washington Medicine Community Health Partner, Kennewick, Washington, United States of America; 2 Department of Cardiology, Lourdes Medical Center, Pasco, Washington, United States of America; 3 Department of Infectious Diseases, Trios Health- A University of Washington Medicine Community Health Partner, Kennewick, Washington, United States of America; Boston Children’s Hospital, UNITED STATES

## Abstract

**Background:**

Research surrounding COVID-19 (coronavirus disease 2019) is rapidly increasing, including the study of biomarkers for predicting outcomes. There is little data examining the correlation between serum albumin levels and COVID-19 disease severity. The purpose of this study is to evaluate whether admission albumin levels reliably predict outcomes in COVID-19 patients.

**Methods:**

We retrospectively reviewed 181 patients from two hospitals who had COVID-19 pneumonia confirmed by polymerase chain reaction (PCR) testing and radiologic imaging, who were hospitalized between March and July 2020. We recorded demographics, COVID-19 testing techniques, and day of admission labs. The outcomes recorded included the following: venous thromboembolism (VTE), acute respiratory distress syndrome (ARDS), intensive care unit (ICU) admission, discharge with new or higher home oxygen supplementation, readmission within 90 days, in-hospital mortality, and total adverse events. A multivariate modified Poisson regression analysis was then performed to determine significant predictors for increased adverse events in patients with COVID-19 pneumonia.

**Results:**

A total of 109 patients (60.2%) had hypoalbuminemia (albumin level < 3.3 g/dL). Patients with higher albumin levels on admission had a 72% decreased risk of developing venous thromboembolism (adjusted relative risk [RR]:0.28, 95% confidence interval [CI]:0.14–0.53, p<0.001) for every 1 g/dL increase of albumin. Moreover, higher albumin levels on admission were associated with a lower risk of developing ARDS (adjusted RR:0.73, 95% CI:0.55–0.98, p = 0.033), admission to the ICU (adjusted RR:0.64, 95% CI:0.45–0.93, p = 0.019), and were less likely to be readmitted within 90 days (adjusted RR:0.37, 95% CI:0.17–0.81, p = 0.012). Furthermore, higher albumin levels were associated with fewer total adverse events (adjusted RR:0.65, 95% CI:0.52–0.80, p<0.001).

**Conclusions:**

Admission serum albumin levels appear to be a predictive biomarker for outcomes in COVID-19 patients. We found that higher albumin levels on admission were associated with significantly fewer adverse outcomes, including less VTE events, ARDS development, ICU admissions, and readmissions within 90 days. Screening patients may lead to early identification of patients at risk for developing in-hospital complications and improve optimization and preventative efforts in this cohort.

## Introduction

Since the start of the pandemic, there have been incredible efforts to study coronavirus disease 2019 (COVID-19), including public health prevention [1], advancing diagnostic capabilities [2], treatment and vaccine development [3], and understanding the broad spectrum of disease sequelae [4]. While researchers and pharmaceutical industries are currently racing for the development of effective antiviral medications and vaccines, research in the medical management of COVID-19 has also expanded tremendously [5–7]. Because of the broad variety of disease presentation, it is vital to determine predictors of worse outcomes for patients with COVID-19. This is of significant importance because the literature has shown that severe acute respiratory syndrome coronavirus 2 (SARS-CoV-2) can affect multiple organs and result in catastrophic events, such as myocardial infarction [8–10], pulmonary embolism [11], cerebral vascular accidents [12, 13], hepatic injury [14, 15], and mortality [16].

Several studies have identified biomarkers correlated with multiple organ failure and severe systemic disease in COVID-19 patients [17, 18]. In a study by Chen et al. [19], organ-related injury has been observed with subsequent higher elevations of aspartate transaminase (AST), alanine transaminase (ALT), cardiac troponin I, N-terminal pro-brain natriuretic peptide (NT-proBNP), and D-dimer levels in non-survivors compared to recovered patients. Lymphopenia was reported in 25% to 85% of patients with COVID-19 and has been shown to predict disease severity [20–22]. Platelet count, procalcitonin, interleukin-6, C-reactive protein (CRP), erythrocyte sedimentation rate (ESR), serum ferritin, and other laboratory tests have been identified as markers for potential progression to COVID-19 critical illness [18, 23–26].

Albumin is a protein synthesized in the liver that has many physiological functions such as providing oncotic pressure, binding and transporting substances, and maintaining acid-base equilibrium, among other functions [27, 28]. During critical illness, inflammatory mediators decrease albumin synthesis in order to prioritize synthesis of other acute phase reactants. Additionally, these mediators increase vascular permeability allowing albumin to escape to the extravascular space, which may also lead to low serum albumin levels [29]. Moreover, low serum albumin concentrations in critical illness have been associated with poor outcomes [30, 31]. A meta-analysis of 90 cohort studies with acutely ill patients by Vincent et al. [32] showed that for each 1 g/dL decline in serum albumin concentration there was a significant rise in the odds of mortality by 137%, morbidity by 87%, and prolonged hospital stay by 71%. Studies have also demonstrated an inverse relationship between serum albumin levels and risk of developing venous thromboembolism (VTE) [33, 34]. Although the field of COVID-19 research is rapidly growing, few studies have examined the association between albumin levels and COVID-19 disease severity [35–37].

The purpose of our study is to identify whether there is an association between initial admission albumin levels and adverse outcomes in COVID-19 patients. Understanding the implications of hypoalbuminemia will help clinicians improve patient care by taking necessary precautions to prevent hospital complications. We aim to investigate independent demographic variables and initial laboratory data to help identify patients with adverse outcomes, including: VTE, acute respiratory distress syndrome (ARDS), intensive care unit (ICU) admission, readmission within 90 days, discharge with new or higher home oxygen supplementation, and in-hospital mortality.

## Materials and methods

**Fig 1:**
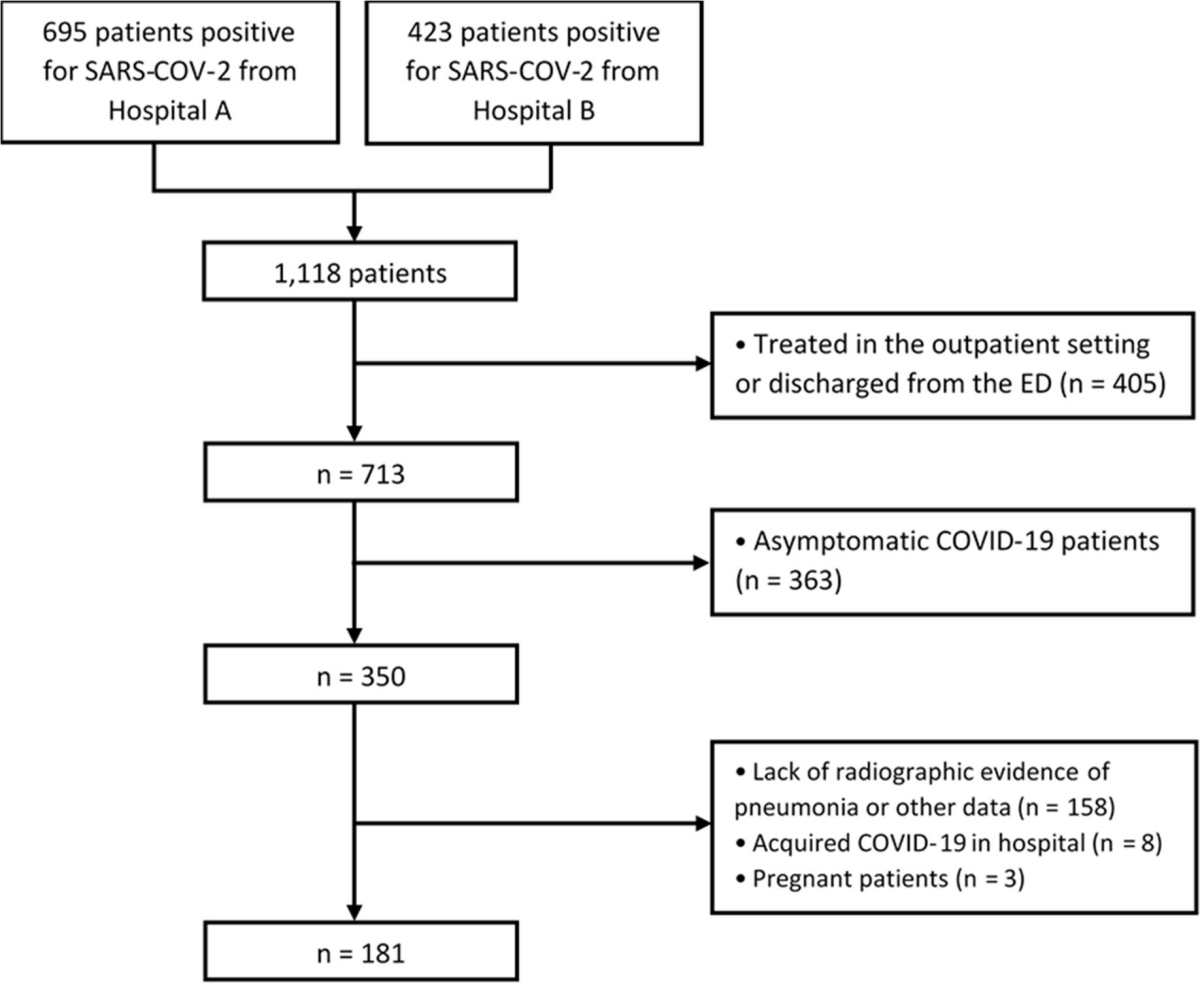
Flowchart of inclusion and exclusion criteria.

We retrospectively reviewed the charts of all patients in two community hospitals from March to July 2020. We identified a total of 1,118 patients (695 patients from Hospital A, and 423 patients from Hospital B) who were confirmed to be positive for COVID-19. Patients were included in the study if they were above 18 years of age, admitted as an inpatient, tested positive for SARS-CoV-2 by nucleic acid amplification (NAA) during that admission, showed COVID-19 symptomatology (e.g., fever, chills, cough, dyspnea, GI disturbances, etc.), exhibited radiographic findings suggestive of viral pneumonia, and if the ED physician and hospitalist physician made a consensus agreement on the diagnosis of COVID-19 pneumonia. We excluded patients who were treated as an outpatient or only in the emergency department (n = 405), asymptomatic carriers of SARS-CoV-2 (n = 363), patients presenting with only upper respiratory symptoms without radiographic evidence of pneumonia (n = 158), patients who acquired COVID-19 during their hospitalization (n = 8), or pregnant patients (n = 3). This left us with our final cohort of 181 patients who met our inclusion criteria (Fig 1).

This study was approved by the Western Institutional Review Board (WIRB) and no external funding was received for this study. Western Institutional Review Board (WIRB) approved a request for a waiver of authorization for use and disclosure of protected health information (PHI) for our research. This review was conducted through expedited review. Waiver of the Health Insurance Portability and Accountability Act (HIPAA) authorization as well as waiver of written informed consent were granted as the data were analyzed anonymously. A team of physicians retrospectively reviewed clinical electronic medical records, comprising of clinical notes and laboratory values for our cohort of patients. We recorded the following demographic data: age, sex, and body mass index (BMI). In terms of laboratory values, initial values on the day of admission were collected, including albumin, AST, ALT, and absolute lymphocyte counts. Hypoalbuminemia was defined as an albumin level < 3.3 g/dL, while a value above this was considered as normal. Outcomes recorded included: VTE events, development of ARDS, ICU admissions, in-hospital mortality events, discharges with new or higher home oxygen supplementation, 90-day readmissions, and total adverse events (sum of outcomes detailed above).

### Statistical analysis

We used the statistical software packages R (version 3.6.1, http://www.R-project.org, The R Foundation) to conduct all the statistical analyses. Categorical variables were presented as frequencies and percentages, and continuous variables as means and standard deviations. Univariate analyses were conducted using Student *t* tests, Pearson Chi-square tests, as well as Fisher’s exact test to compare groups of patients with hypoalbuminemia and normal albumin levels in order to identify variables significantly associated with adverse outcomes. Using multivariate modified Poisson regression [38], we estimated the relative risk for the effects of several variables on the aforementioned outcomes and 95% confidence intervals (CIs) were reported. An alpha level of 0.05 was considered statistically significant. Data analysis was performed in January 2021.

## Results

### Univariate analysis

After exclusions, our study involved 181 patients, of which 97 (53.6%) patients were male and the average age was 65 years (standard deviation [SD]: 19.1 years). The average albumin level in the cohort was 3.17 g/dL (SD: 0.66 g/dL) and a total of 109 patients (60.2%) had hypoalbuminemia. Based on our univariate analyses, patients were more likely to have higher albumin levels if they were younger (p < 0.001). Higher albumin levels were also associated with lower in-hospital mortality (p = 0.020) and fewer total adverse outcomes (p < 0.001; Table 1). Mean albumin levels were higher for patients who were not admitted to the ICU (3.25 g/dL) compared to those that were (2.93 g/dL) (p = 0.008). Patients admitted to the ICU were more likely to have higher total adverse outcomes (p < 0.001) including VTE events (p = 0.032), ARDS (p < 0.001), and in-hospital mortality (p < 0.001; Table 2).

**Table 1:** Characteristics of the patient cohort with and without hypoalbuminemia.

		Hypoalbuminemia (albumin < 3.3)	Normal Albumin (albumin ≥ 3.3)	P-Value
**Age (mean = 65.0; SD = 19.1)**		68.9	59.2	< 0.001*
**Sex**	Female	50 (0.60)	34 (0.40)	0.859
	Male	59 (0.61)	38 (0.39)	
**BMI (mean = 31.5; SD = 7.88)**		30.8	32.4	0.179
**Albumin (mean = 3.17; SD = 0.661)**		2.75	3.82	< 0.001*
**AST/ALT (mean = 1.35; SD = 0.552)**		1.40	1.27	0.117
**Lymphocytes (mean = 1.06; SD = 0.581)**		1.03	1.12	0.289
**VTE**	Yes	10	1	0.052¶
	No	99	71	
**ARDS**	Yes	43	19	0.070
	No	66	53	
**ICU Admission**	Admitted	30	16	0.423
	Not admitted	79	56	
**In-hospital Mortality**	Alive	74	60	0.020*
	Dead	35	12	
**Discharged with Home O2**	With Home O2	30	17	0.557
	Without	79	55	
**90-Day Readmission**	Readmission	11	2	0.079
	No readmission	98	70	
**Total Adverse Outcomes**	0	28	36	< 0.001*
	1	21	16	
	2	31	9	
	3	17	11	
	4	11	0	
	6	1	0	

\BMI: Body Mass Index. AST: Aspartate Transaminase. ALT: Alanine Transaminase. VTE: Venous Thromboembolism. ARDS: Acute Respiratory Distress Syndrome. ICU: Intensive Care Unit. O2: Oxygen. “Total adverse events” is defined as the summation of the following events: VTE, ARDS, ICU admission, in-hospital mortality, discharge with home O2, and 90-day readmission.

* Indicates P-value < 0.05.

¶ Fisher’s exact test used.

**Table 2:** Characteristics of the patient cohort with and without ICU admission.

		No ICU admission	ICU admission	P-Value
**Age (mean = 65.0; SD = 19.1)**		65.3	64.4	0.740
**Sex**	Female	63 (0.75)	21 (0.25)	0.905
	Male	72 (0.74)	25 (0.26)	
**BMI (mean = 31.5; SD = 7.88)**		31.1	32.6	0.210
**Albumin (mean = 3.17; SD = 0.661)**		3.25	2.93	0.008*
**AST/ALT (mean = 1.35; SD = 0.552)**		1.29	1.52	0.035*
**Lymphocytes (mean = 1.06; SD = 0.581)**		1.10	0.970	0.175
**VTE**	Yes	5	6	0.032*¶
	No	130	40	
**ARDS**	Yes	20	42	< 0.001*
	No	115	4	
**In-hospital Mortality**	Alive	110	24	< 0.001*
	Dead	25	22	
**Discharged with Home O2**	With Home O2	34	13	0.681
	Without	101	33	
**90-Day Readmission**	Yes	9	4	0.741¶
	No	126	42	
**Total Adverse Outcomes**	0	64	0	< 0.001*¶
	1	37	0	
	2	31	9	
	3	3	25	
	4	0	11	
	6	0	1	

ICU: Intensive Care Unit. BMI: Body Mass Index. AST: Aspartate Transaminase. ALT: Alanine Transaminase. VTE: Venous Thromboembolism. ARDS: Acute Respiratory Distress Syndrome. O2: Oxygen. “Total adverse events” is defined as the summation of the following events: VTE, ARDS, ICU admission, in-hospital mortality, discharge with home O2, and 90-day readmission.

* Indicates P-value < 0.05.

¶ Fisher’s exact test used.

### Multivariate analysis

Multivariate modified Poisson regression analyses were performed, and risk ratios (RR) were calculated after adjusting for age, sex, and BMI. It was found that patients with higher albumin levels on admission have 72% decreased risk of developing VTE (adjusted RR: 0.28, 95% CI: 0.14–0.53, p < 0.001) for every 1 g/dL increase of albumin. There was a lower risk of developing ARDS (adjusted RR: 0.73, 95% CI: 0.55–0.98, p = 0.033) with higher albumin levels on admission. Moreover, the higher the albumin on admission, the less likely the patient was admitted to the ICU (adjusted RR: 0.64, 95% CI: 0.45–0.93, p = 0.019). Patients with higher initial albumin values were less likely to be readmitted within 90 days (adjusted RR: 0.37, 95% CI: 0.17–0.81, p = 0.012). Furthermore, higher albumin was associated with fewer total adverse events (adjusted RR: 0.65, 95% CI: 0.52–0.80, p < 0.001). The remainder of the multivariate analyses can be found in Table 3.

**Table 3:** Multivariate analysis.

Outcomes	VTE		ARDS		ICU Admission		In-hospital Mortality		Discharged with Home O2		Readmitted within 90 Days		Total Adverse Events	
	RR	95% CI	RR	95% CI	RR	95% CI	RR	95% CI	RR	95% CI	RR	95% CI	RR	95% CI
Variables														
**Age**	0.99	(0.97, 1.02)	1.01	(1.00, 1.02)	1.00	(0.98, 1.01)	1.04*	(1.03, 1.06)	1.00	(0.98, 1.01)	0.99	(0.96, 1.02)	1.01*	(1.00, 1.02)
**Sex**	0.89	(0.30, 2.62)	1.08	(0.73, 1.60)	1.11	(0.67, 1.82)	0.76	(0.49, 1.19)	0.74	(0.45, 1.23)	1.56	(0.55, 4.47)	1.00	(0.77, 1.30)
**BMI**	0.99	(0.90, 1.09)	1.01	(0.98, 1.04)	1.03	(0.99, 1.06)	1.02	(0.98, 1.06)	1.03	(1.00, 1.06)	0.98	(0.89, 1.09)	1.01	(1.00, 1.03)
**Albumin**	0.28*	(0.14, 0.53)	0.73*	(0.55, 0.98)	0.64*	(0.45, 0.93)	0.79	(0.57, 1.09)	0.75	(0.50, 1.12)	0.37*	(0.17, 0.81)	0.65*	(0.52, 0.80)
**AST/ALT**	0.66	(0.26, 1.72)	1.60*	(1.18, 2.17)	1.53*	(1.03, 2.27)	1.95*	(1.35, 2.83)	0.60*	(0.37, 0.98)	0.85	(0.27, 2.72)	1.27*	(1.01, 1.59)
**Lymphocytes**	0.46	(0.06, 3.32)	0.96	(0.66, 1.40)	0.81	(0.50, 1.31)	0.91	(0.63, 1.31)	0.83	(0.49, 1.38)	1.74	(0.68, 4.45)	0.97	(0.77, 1.23)

RR: Relative Risk. BMI: Body Mass Index. AST: Aspartate Transaminase. ALT: Alanine Transaminase. VTE: Venous Thromboembolism. ARDS: Acute Respiratory Distress Syndrome. ICU: Intensive Care Unit. O2: Oxygen. “Total adverse events” is defined as the summation of the following events: VTE, ARDS, VTE, ICU admission, in-hospital mortality, discharge with home O2, and 90-day readmission.

*Indicates P-value < 0.05.

## Discussion

With the recent increase in COVID-19 cases in the United States [39], we have a strong impetus to screen patients and risk-stratify patients in an effort to triage those who are predicted to poorly progress. Reliable biomarkers can aid in the prediction of prognosis and allow timely preventative efforts and optimization of high-risk patients. The results of our study have demonstrated that serum albumin levels are predictive of adverse outcomes in patients with confirmed COVID-19. We found that higher albumin levels were associated with better outcomes, including fewer VTE events, ARDS development, ICU admissions, readmissions within 90 days, and total adverse events.

Our findings are consistent with prior studies that demonstrate a correlation between hypoalbuminemia and COVID-19 severity [35–37]. Chen et al. in a study of 88 critically ill patients with COVID-19 noted that low albumin levels, as well as high SOFA scores and elevated D-dimer, were independent risk factors for deep vein thrombosis [35]. Aziz et al. performed a meta-analysis of four studies that demonstrated an increased risk for severe COVID-19 (defined as respiratory distress, ICU admission, and/or death) with hypoalbuminemia [36]. In a retrospective cohort study of 299 patients, Huang et al. showed that hypoalbuminemia was an independent predictor for mortality in COVID-19 patients [37]. Our current study differs from these, however, in that our patient cohort includes only hospitalized patients who had symptomatic COVID-19 and radiographic evidence of pneumonia. In addition, we examined several other relevant prognostic outcomes including discharge with home oxygen supplementation, readmission within 90 days, and total adverse events. Of note, although albumin was associated with in-hospital mortality in our univariate analysis, after controlling for confounders of age, sex, and BMI, the multivariate analysis did not demonstrate a statistically significant association. However, this may be a result of our study restricting the definition of mortality to only include in-hospital mortality whereas the prior studies comparing albumin levels and COVID-19 outcomes did not define restrictions on mortality [36, 37].

Serum albumin concentration can be decreased by a myriad of factors including malnourishment, inflammation, hepatocellular injury, and renal losses [27, 40]. During a state of critical illness, albumin is synthesized less in addition to being extravasated into the interstitial space due to capillary leakage [41]. Nevertheless, adequate albumin levels appear to be crucial since albumin has been shown to display significant antioxidant properties such as scavenging oxygen free radicals (OFR) [42]. Reducing oxidative stress caused by OFR is critical during sepsis since these oxygen free radicals can result in tissue ischemia, reperfusion injury, and an intense systemic inflammatory response [43]. This implies that patients with hypoalbuminemia may have a diminished immunological response to oxygen radical scavenging that occurs during sepsis as well as sepsis-associated complications such as VTE, ARDS, and mortality [44, 45]. Moreover, albumin exhibits anticoagulant effects due to its capability to bind to antithrombin, similar to heparin, as well as its inhibitory effect on platelet aggregation [46]. Consequently, hypoalbuminemia may be a contributing factor to the high incidence of VTE of about 11% to 70% seen in COVID-19 patients [47]. Therefore, in addition to prior known biomarkers (e.g., procalcitonin, CRP, lymphocyte count, D-dimer, troponin I, AST, ALT, among others) associated with severe COVID-19 [18], serum albumin levels might help in prognostic risk stratification.

Limitations of this study include that it was restricted to a single geographic region setting and a retrospective design. In addition, patients with presumed COVID-19 who tested negative for SARS-CoV-2 NAA were not included in the study. Other than obesity, the other comorbidities of the patients were not taken into account in the analysis. Comorbidities associated with low serum albumin levels such as cirrhosis, chronic kidney disease, end-stage renal disease, nephrotic syndrome, protein-losing enteropathy, and chronic protein-calorie malnutrition [40] were not adjusted for in this article. Moreover, this study took into consideration the albumin level on admission only. Perhaps the final albumin value or the change in albumin values are predictors of disease severity as well. Even so, despite these limitations we consider these findings valuable to health care providers treating these patients.

In conclusion, COVID-19 patients with higher albumin levels on admission were associated with a better overall prognosis. Higher albumin levels appear to confer a lower risk of VTE events. Therefore, serum albumin level may aid in decisions regarding VTE chemoprophylaxis early in the hospitalization. In addition, due to the link between albumin levels and ICU admissions as well as ARDS development, patients with severe hypoalbuminemia may benefit from admission to a higher level of care (e.g., intermediate care) compared to the general medical floor if available. Patients with lower albumin levels may also benefit from strategies aimed at reducing readmissions (e.g., providing transitional care, ensuring close follow-up, addressing clear post-discharge instructions, etc.). Further investigation is warranted to demonstrate if the correction of hypoalbuminemia on admission, through methods such as intravenous albumin administration or early protein supplementation, will improve outcomes for COVID-19 patients.
